# Silymarin protects liver against toxic effects of anti-tuberculosis drugs in experimental animals

**DOI:** 10.1186/1743-7075-5-18

**Published:** 2008-07-05

**Authors:** Sude Eminzade, Fikriye Uras, Fikret V Izzettin

**Affiliations:** 1 Department of Pharmacology, Marmara University, Faculty of Pharmacy, Haydarpasa, Istanbul, Turkey; 2 Department of Biochemistry, Marmara University, Faculty of Pharmacy, Haydarpasa, Istanbul, Turkey

## Abstract

**Background:**

The first line anti-tuberculosis drugs isoniazid (INH), rifampicin (RIF) and pyrazinamide (PZA) continues to be the effective drugs in the treatment of tuberculosis, however, the use of these drugs is associated with toxic reactions in tissues, particularly in the liver, leading to hepatitis. Silymarin, a standard plant extract with strong antioxidant activity obtained from *S. marianum*, is known to be an effective agent for liver protection and liver regeneration. The aim of this study was to investigate the protective actions of silymarin against hepatotoxicity caused by different combinations of anti-tuberculosis drugs.

**Methods:**

Male Wistar albino rats weighing 250–300 g were used to form 6 study groups, each group consisting of 10 rats. Animals were treated with intra-peritoneal injection of isoniazid (50 mg/kg) and rifampicin (100 mg/kg); and intra-gastric administration of pyrazinamid (350 mg/kg) and silymarin (200 mg/kg). Hepatotoxicity was induced by a combination of drugs with INH+RIF and INH+RIF+PZA. Hepatoprotective effect of silymarin was investigated by co-administration of silymarin together with the drugs. Serum biochemical tests for liver functions and histopathological examination of livers were carried out to demonstrate the protection of liver against anti-tuberculosis drugs by silymarin.

**Results:**

Treatment of rats with INH+RIF or INH+RIF+PZA induced hepatotoxicity as evidenced by biochemical measurements: serum alanine aminotransferase (ALT), aspartate aminotransferase (AST) and alkaline phosphatase (ALP) activities and the levels of total bilirubin were elevated, and the levels of albumin and total protein were decreased in drugs-treated animals. Histopathological changes were also observed in livers of animals that received drugs. Simultaneous administration of silymarin significantly decreased the biochemical and histological changes induced by the drugs.

**Conclusion:**

The active components of silymarin had protective effects against hepatotoxic actions of drugs used in the chemotherapy of tuberculosis in animal models. Since no significant toxicity of silymarin is reported in human studies, this plant extract can be used as a dietary supplement by patients taking anti-tuberculosis medications.

## Background

Rifampicin (RIF), isoniazid (INH), pyrazinamid (PZA) and ethambutol are first line drugs used for the treatment of tuberculosis. Rifampicin has bactericidal activity against *M. tuberculosis *by inhibiting bacterial DNA-dependent RNA polymerase [1]. Isoniazid is a prodrug activated by bacterial catalase-peroxidase (KatG) and kills actively growing tubercle bacilli by inhibiting the biosynthesis of mycolic acids which are major components of cell wall of *M. tuberculosis *[1,2]. The other prodrug, pyrazinamid, is activated by bacterial pyrazinamidinase which is only active in acidic conditions (pH: 5.5). The active metabolite is pyrazinoic acid that inhibits fatty acid synthesis in *M. tuberculosis *[3]. This drug is used in the initial two months of treatment to reduce the duration of therapy, and is not used alone [4]. Ethambutol inhibits the synthesis of some metabolites in actively growing *M. tuberculosis*, causing impairment of cell metabolism, arrest of multiplication, and cell death [5].

Drugs are not used solely in the treatment of tuberculosis. Instead, the first line drugs are used in combination, or with other medicines. The single use of drug may result in the rapid development of resistance or failure of treatment. Several regimens are available for the treatment of tuberculosis. Depending on the duration of treatment or in the case of resistance, individual drugs may be omitted from the protocol. Several adverse reactions of anti-tuberculosis drugs are reported. The best known toxic drug effect is hepatotoxicity. The frequency and severity of hepatotoxicity is increased when these drugs are used in combination [1,4,6]. Anti-tuberculosis drugs act as inducers of hepatic cytochrome P450 enzymes. For example, rifampicin is a potent inducer of CYP2D6 and CYP3A4, and isoniazid induces CYP2E1 [6,7]. The induction of CYT P450 enzymes is known to take part in increased drug disposition and development of multi-drug resistance. Xenobiotics, including anti-tuberculosis drugs, undergo biotransformation in the liver catalyzed by microsomal enzyme systems. The major isozyme of cytochrom P450 enzymes in bioactivation is CYT2E1, which is also involved in hepatic toxicity of carbon tetrachloride, ethanol and acetaminophen. Inhibition of this isozyme by specific inhibitors or herbal drugs has been shown to be hepatoprotective [8-10]. Several reactive derivatives of drugs and oxidants are generated during the process of drug biotransformation. The reactive species generated can bind and/or react with cellular components in the liver, and cause liver injury resulting in impairment of liver functions. Reaction of reactive species with cellular antioxidants causes antioxidant depletion that may result in oxidative stress [9,11,12].

Recent studies indicate the existence of a strong correlation between hepatic injury and oxidant stress in experimental animals treated with anti-tuberculosis drugs [8,12-17]. Since all the drugs used in the treatment of tuberculosis are shown to have hepatotoxic effects, studies have been performed to prevent or reduce the toxicity by the use of natural herbal drugs and/or synthetic compounds, without interfering with the therapeutic actions of the drugs. Garlic [14], silymarin, [12,15] N-acetylcysteine [16,17] and several other herbal drugs are proved to have such effects. It is of importance to note that the inhibition of CYTP450 2E1 and antioxidant actions seem to be the common mechanism of action of herbal drugs [9-15]. Among the herbal drugs, silymarin has been used as a dietary supplement for hepatoprotection for over 2000 years. Silymarin, commercially avaible as Milk Thistle, is an extract from the seeds of *S. Marianum*. Silybines (A and B isomers), isosilybines (A and B), silychristine and silydianine are active flavonoids found in silymarin extract [18]. Silymarin has been shown to be safe in animal models, and no significant adverse reactions are reported in human studies [18]. In this study, we researched the hepatoprotective effects of silymarin in rats treated with two different combinations of antituberculosis drugs isoniazid, rifampicin and pyrazinamid.

## Methods

### Animals

Male Wistar albino rats weighing about 250–300 g were selected and used in the present study. All experiments were carried out according to the guidelines for the care and use of experimental animals, and approved by the Animals Ethical Committee of Marmara University Faculty of Medicine. Rats were acclimatized for 2 weeks before starting the experiments. Animals were maintained in a 12 h light/dark cycle at 25°C and were free access to standard pellet food and tap water. One way variance analysis showed no significant difference between the mean weight of animals at the beginning of the treatment (mean weight of each group was 275 g with a standard deviation of 13). Intake of feed and water was observed and assessed for all the animals during the treatment.

### Drugs and Chemicals

The first line anti-tuberculosis drugs used in the study were isoniazid (from ZheJiang TaiZhou JiangBei chemical factory), rifampicin (CKD Bio Corporation) and pyrazinamid (Koçak Medicinal Industry). Milk Thistle (silymarin) was purchased from Solgar. Other chemicals used in the study were of analytic grade. Isoniazid and rifampicin were separately dissolved in sterile distilled water. Because of poor solubility, suspensions of pyrazinamid and silymarin were prepared for intra-gastric administration [19].

### Animal Study Protocol

Sixty animals used in this study were divided into six groups. Blood samples were taken from the tail vein of each animal to evaluate liver functions before the treatment. The first group (Group I) served as the control group, and was intra-peritonealy injected with sterile saline (1 ml/kg). The second group (Group II) was silymarin-control group that received intra-gastric administration of silymarin suspension at a dose of 200 mg/kg (silymarin suspension was prepared by suspending the content of Milk Thistle gelatin capsules in sterile distilled water). The third (Group III) and fourth (Gourp IV) groups were hepatotoxicity model groups that were intra-peritoneally injected with isoniazid (50 mg/kg) and rifampicin (100 mg/kg) [12,13,16,20]. The fourth group of animals received 350 mg/kg pyrazinamid via intra gastric route in addition to isoniazid and rifampicin. The fifth (group V) and sixth (Group VI) groups received the same drugs at the same doses as given for Group III and Group IV, respectively; except that the last two groups were treated additionally with intra-gastric administration of silymarin at a dose of 200 mg/kg. Drugs were given once daily according to the above protocol over 14 days. One day after the last treatment, body weights were recorded, and animals were anesthesized with intra-peritoneal injection of high dose ketamin. Blood was obtained by cardiac puncture immediately, and livers were saved for histopathological examination.

### Biochemical Assessments

The liver is the site of bilirubin detoxification and excretion. Several plasma proteins, including albumin, are synthesized in the liver, and the liver also regulates plasma lipids and lipoproteins. Therefore, the levels of albumin, total protein and bilirubin can be used as indicators of liver function. Serum ALT, AST and ALP activities are considered as good markers of hepatic injury and hepatocellular integrity. We measured the activities of these enzymes before starting the treatment (as baseline values) and after the treatment of rats with the drugs.

Blood samples obtained either from tail vein (before treatments) or cardiac puncture (after the treatment) were centrifuged and serum samples were stored in a deep freezer until they could be analyzed. Serum alanine aminotransferase (ALT), aspartate aminotransferase (AST), alkaline phosphatase (ALP) activities and the levels of serum albumin, serum total protein, and serum total bilirubin were determined by Hitachi-917 auto-analyzer using kits manufactured by Roche Diagnostic Division.

### Histopathological Investigation

Small pieces of liver tissues from animals were preserved in 10% formaldehyde, and later were embedded into paraffin blocks. Thin sections (5 μM) were prepared from the blocks for microscopic examination. Light microscopy was performed after slides were routinely stained with haematoxylin and eosin (H&E).

### Statistical Analysis

In biochemical determinations, each sample was assayed in duplicate, and average values were calculated as means plus or minus standard deviation (SD). Data were analyzed by one-way analysis of variance (ANOVA), followed by multiple comparisons using Dunnett's procedure to compare all groups against control. Unpaired Student *t *testing was also used to assess differences between groups. A *P *value of less than 0.05 was considered statistically significant.

## Results

Before the initiation of the drug treatment, baseline liver function tests were performed and any animal with abnormal liver function test was excluded from the study. Water and food intake was the same in all groups and no mortality was observed throughout the study. Body weights were increased in all groups as compared with the weights before the treatment. However, no significant difference in body weights was observed between the groups. Similar results are reported in earlier studies [1,2].

**Figure 1:**
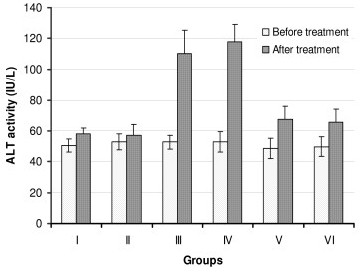
**Serum ALT activities of rats before and after treatment with anti-tuberculosis drugs and silymarin**. Constant doses of INH (50 mg/kg), RIF (100 mg/kg), PZA (350 mg/kg) and silymarin (200 mg/kg) were used. Group I: Control, Group II: Silymarin control, Group III: INH+RIF, Group IV: INH+RIF+PZA, Group V: INH+RIF+Silymarin, and Group VI: INH+RIF+PZA+Silymarin. * *P *< 0.001 as compared with groups I, II, V and VI. * *P *< 0.05 as compared with Groups I and II.

**Figure 2:**
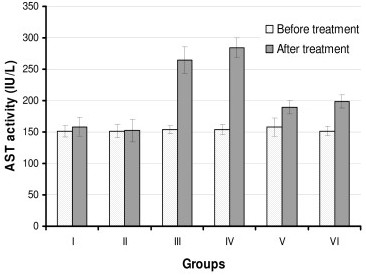
**Serum AST activities of rats before and after treatment with anti-tuberculosis drugs and silymarin**. Constant doses of INH (50 mg/kg), RIF (100 mg/kg), PZA (350 mg/kg) and silymarin (200 mg/kg) were used. Group I: Control, Group II: Silymarin control, Group III: INH+RIF, Group IV: INH+RIF+PZA, Group V: INH+RIF+Silymarin, and Group VI: INH+RIF+PZA+Silymarin. * *P *< 0.001 as compared with groups I, II, V and VI. * *P *< 0.05 as compared with Groups I and II.

**Figure 3:**
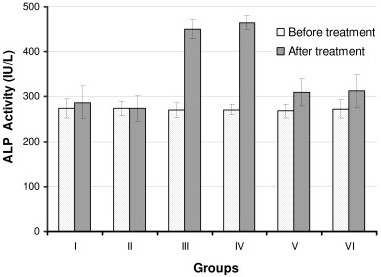
**Serum ALP activities of rats before and after treatment with anti-tuberculosis drugs and silymarin**. Constant doses of INH (50 mg/kg), RIF (100 mg/kg), PZA (350 mg/kg) and silymarin (200 mg/kg) were used. Group I: Control, Group II: Silymarin control, Group III: INH+RIF, Group IV: INH+RIF+PZA, Group V: INH+RIF+Silymarin, and Group VI: INH+RIF+PZA+Silymarin. * *P *< 0.001 as compared with groups I, II, V and VI. * *P *< 0.05 as compared with Groups I and II.

We measured the activities of ALT, AST and ALP before starting the treatment (as baseline values) and after the treatment of rats with the drugs. The serum ALT, AST and ALP activities of the control group (group I) and the silymarin-control group (group II) did not show any significant difference when compared with baseline values (Figures 1, 2, 3). However, treatment of rats with INH + RIF (group III) or with INH + RIF + PZA (group IV) caused a 2 fold increase (*P *< 0.001) from their baseline of ALT (Figure 1). The activities of serum AST and ALP were also increased (*P *< 0.001) approximately 75% in drugs-treated animals, indicating that the drugs had caused a decrease in hepatocellular integrity (Figures 2 and 3). Pyrazinamide did not seem to exacerbate the hepatotoxic actions of INH and RIF when serum enzyme activities are evaluated (Figures 1, 2, 3, *P *> 0.05).

Simultaneous intra-gastric administration of silymarin with anti-tuberculosis drugs either with INH + RIF (group V) or INH + RIF + PZA (group VI) significantly decreased (*P *< 0.001) the serum enzyme activities when compared with hepatotoxicity groups (Groups III and IV).

We measured serum albumin, total protein and total bilirubin levels in rats before and after the treatment with the drugs. Treatment of rats with INH + RIF or INH + RIF + PZA significantly decreased (*P *< 0.001) serum albumin and total protein concentrations and caused a 2 fold increase in total bilirubin levels (Tables 1 and 2). No significant difference was found between group III and group IV, indicating that PZA did not aggravate the toxic effects of INH and RIF. Co-administration of silymarin with INH + RIF or with INH + RIF + PZA significantly increased serum albumin and total protein concentrations and decreased serum total bilirubin levels when compared with rat groups that received drugs only (groups III and IV) (*P *< 0.001). However, drugs and silymarin-treated groups still had lower albumin and total protein, and higher total bilirubin levels when compared with the control (*P *< 0.01), except that total protein of group VI was not different from that of control (Table 2).

**Table 1:** Baseline levels of serum albumin, total protein and bilirubin in rats before treatment with anti-tuberculosis drugs.

Groups	Albumin (gr/dl)	Total protein (gr/dl)	Total bilirubin (mg/dl)
I (control)	4.51 ± 0.30	7.56 ± 0.43	0.077 ± 0.015
II (silymarin control)	4.65 ± 0.37	7.89 ± 0.41	0.080 ± 0.014
III (INH+RIF)	4.57 ± 0.33	7.81 ± 0.33	0.086 ± 0.016
IV (INH+RIF+PZA)	4.75 ± 0.32	7.78 ± 0.58	0.085 ± 0.019
V (INH+RIF + Silymarin)	4.73 ± 0.38	7.70 ± 0.56	0.077 ± 0.018
VI (INH+RIF+PZA+Silymarin)	4.47 ± 0.31	7.66 ± 0.31	0.096 ± 0.017

Values represent mean ± SD.

**Table 2:** The levels of albumin, total protein and total bilirubin after the treatment of rats with antituberculosis drugs and silymarin.

Groups	Albumin (gr/dl)	Total Protein (gr/dl)	Total Bilirubin (mg/dl)
I (control)	4.20 ± 0.40	7.27 ± 0.32	0.095 ± 0.022
II (silymarin control)	4.42 ± 0.40	7.49 ± 0.34	0.099 ± 0.021
III (INH+RIF)	3.44 ± 0.32*	5.80 ± 0.59*	0.194 ± 0.052*
IV (INH+RIF+PZA)	3.35 ± 0.17*	5.70 ± 0.48*	0.199 ± 0.049*
V (INH+RIF+Silymarin)	4.02 ± 0.28**	6.84 ± 0.41**	0.118 ± 0.036**
VI (INH+RIF+PZA+Silymarin)	3.95 ± 0.47***	6.85 ± 0.59**	0.137 ± 0.026**

Values represent mean ± SD. Constant doses of INH (50 mg/kg), RIF (100 mg/kg), PZA (350 mg/kg) and silymarin (200 mg/kg) were used. *P < 0.001 as compared to the controls (Groups I and II). ** *P *< 0.01 as compared to the controls (Groups I and II) and hepatotoxicity groups (Groups II and IV). *** *P *< 0.05 significant difference from the hepatotoxicity groups but not the control.

Light microscopic histological evaluation of liver tissues is given in Table 3. The control and the silymarin group animals had normal liver histology. Liver degeneration was observed in all animals from groups III and IV. In group V and VI, 6 out of 10 rats had degenerated liver. Steatosis and patchy necrosis was detected in hepatotoxicity groups (Groups III and IV), and the frequency of both steatosis and patchy necrosis was decreased by silymarin administration. Portal triaditis was detected in group III and group IV, which were not detectable in groups receiving drugs plus silymarin (groups V and VI). Despite earlier findings [2], we did not observe any fibrosis or regeneration in the liver of any rat (Table 3).

**Table 3:** Histological evaluation of liver tissues by light microscopy.

	Group I	Group II	Group III	Group IV	Group V	Group VI
Degeneration	0/10	0/10	**10/10**	**10/10**	**6/10**	**6/10**
Steatosis	0/10	0/10	**7/10**	**7/10**	**3/10**	**2/10**
Necrosis	0/10	0/10	**7/10**	**8/10**	**2/10**	**2/10**
Triaditis	0/10	0/10	**8/10**	**8/10**	0/10	0/10
Fibrosis	0/10	0/10	0/10	0/10	0/10	0/10
Regeneration	0/10	0/10	0/10	0/10	0/10	0/10

Constant doses of INH (50 mg/kg), RIF (100 mg/kg), PZA (350 mg/kg) and silymarin (200 mg/kg) were used. Group I: Control, Group II: Silymarin control, Group III: INH+RIF, Group IV: INH+RIF+PZA, Group V: INH+RIF+Silymarin, and Group VI: INH+RIF+PZA+Silymarin.

## Discussion

Tuberculosis is a leading public health problem world wide, particularly in developing countries. About one third of world's population has latent tuberculosis and approximately 9 million cases of active tuberculosis emerge annually resulting in 2–3 million deaths [20]. Out of 1.86 billion people estimated to be infected with the tuberculosis bacillus, an estimated 1.3 billion infected people were living in developing countries, such as India and China [21]). In view of the seriousness of the problem World Health Organization (WHO) declared it to be a global emergency in 1993. Active tuberculosis will kill about two out of every three people if untreated. Even treated tuberculosis has a mortality rate of just less than 5% [22]. Anti-tuberculosis drugs are used in combination, except in latent tuberculosis or chemoprophylaxis. The use of INH and RIF is associated with hepatotoxicity in some individuals. The rate of hepatotoxicity is much higher in developing countries like India (8%–30%) compared to that in advanced countries [23,24].

Rats have been used successfully to investigate INH and RIF-induced hepatotoxicity models [4,12-17]. Therefore, we selected rats to study the hepatotoxic effect of anti-tuberculosis drugs and hepatoprotective action of silymarin. In the present study, hepatotoxicity was produced by daily co-administration of INH+RIF or INH+ RIF+PZA over 14 days. Since hepatotoxic action of INH and RIF is well documented [11-13,16,17,20,25], instead of single use of drugs, we treated the animals with two different combinations as done in the treatment of tuberculosis. The doses of the drugs used (INH: 50 mg/kg, RIF: 100 mg/kg and PZA: 350 mg/kg) are very high compared to those used in the treatment of tuberculosis in human subjects. However, higher doses of drugs are required in animal models to produce hepatotoxicity, because rats metabolize the drugs at a faster rate and the duration of treatment is much shorter compared to the treatment of tuberculosis in humans.

Biochemical tests related to the hepatocellular integrity can be checked to follow hepatocellular integrity and liver injury. In this study, the injection of INH and RIF caused a significant elevation (approximately 2 fold increase) in the activities of ALT, AST and ALP. Increased activity of these enzymes showed that the integrity of hepatocytes was abnormal, resulting in the release of intracellular enzymes into the systemic circulation (Figures 1, 2, 3). Much higher activities of these enzymes is measured in serum when rats are treated with INH and RIF over longer period, up to 90 days [26]. Serum total bilirubin was also increased two fold, indicating membrane damage in the liver (Table 2). Decrease in albumin and total protein levels showed that administration of drugs has caused impairment of liver function, e.g. its capacity to synthesize albumin (Table 2). Histopathological changes observed in drugs-treated animals correlated well with biochemical results (Table 3). Based on *in vivo *and *in vitro *results, it is suggested that the underlying mechanism for hepatotoxicity is related to: *1) *drug bioactivation by CYTP450 2E1, *2) *generation of oxygen free radicals and reactive metabolites of drugs, *3) *imbalance in the oxidant/antioxidant defense, and *4) *eventual peroxidation of membrane lipids that leads to the loss of hepatocellular integrity and failure of liver function. In the present study, inclusion of PZA to the treatment protocol (Group IV) did not further enhance the hepatotoxicity of INH and RIF (Table 4). This result is consistent with earlier observation that PZA did not promote lipid peroxidation, and has no effect on antioxidant status in the liver, either when is used alone or with INH and/or RIF [26].

The available literature shows that the extracts obtained from several plants have hepatoprotective activities against the toxicity induced by xenobiotics, including those that are used in the treatment of tuberculosis [12,14,19,21,27,28]. Silymarin, the root extract from *Silybum marianum*, is known to have such an activity. In this study, the administration of silymarin together with INH+RIF or INH+RIF+PZA decreased hepatotoxicity of drugs as judged from liver function tests. Silymarin decreased serum ALT, AST and ALP activities, and the levels of bilirubin; increased serum albumin and total protein concentration in drugs-treated animals. The serum ALT, AST and ALP activities of the control group (group I) and the silymarin-control group (group II) did not show any significant difference when compared with baseline values. This showed that silymarin has no significant undesired actions on hepatocellular integrity on its own. However, silymarin did not have a complete protection against drug toxicity, since groups V and VI still had higher serum enzyme activities when compared with the controls (*P *< 0.05) (Figures 1, 2, 3).

Silymarin administration significantly reduced the number of animals with pathological liver changes, and the severity of changes were less, if any, compared to the hepatotoxicity groups. Consistent with previous studies, these results clearly show that silymarin has protective effects towards the toxic actions of anti-tuberculosis drugs [12,19]. Recently, several studies have been carried out to elucidate the mechanism of action of silymarin. Accumulated data show that this herbal drug inhibits several isoforms of CYT P450 enzymes [7-10], potentiates the antioxidant capacity of the liver [20], acts as a scavenger of oxygen free radicals [29], inhibits the synthesis of proinflamatory cytokines and enhances apoptosis. In addition to its hepatoprotective actions, silymarin is shown to be effective in other organs including lung and brain; and to inhibit tumor growth and promotion in several types of cancer [18,30].

## Conclusion

This study showed that silymarin has a significant protective action against the hepatotoxicity induced by the drugs used in the treatment of tuberculosis. Although silymarin has been used as a medical intervention for more than 2000 yr, there remains insufficient evidence to support or refuse its use in patients with liver disease. High-quality, placebo-controlled randomized trials need to be conducted before silymarin or its constituents can be advocated as a medicine for use in humans. Since no significant toxicity of silymarin is reported in human studies, this plant extract can be used as a dietary supplement by patients taking anti-tubercular medications.

## Abbreviations

INH: Isoniazid; RIF: Rifampicin; PZA: Pyrazinamid; ALT: Alanine transaminase; AST: Aspartate transaminase; ALP: Alkaline phosphatase.

## Competing interests

The authors declare that they have no competing interests.

## Authors' contributions

FVI and SE conceived and designed the study. SE carried out animal treatments, biochemical experiments, and data analysis. FU participated in biochemical determinations. SE, FU and FVI participated in interpretation of results and preparation of the article. All authors read and approved the final version of this manuscript.
